# Successful treatment of acute organophosphorus pesticide poisoning complicated by intestinal necrosis with exploratory laparotomy: a case report

**DOI:** 10.1515/med-2026-1458

**Published:** 2026-07-14

**Authors:** Shuhong Wang, Lili Shi, Yin Tian, Jin Wu, Xiangui Zhou, Ruilie Wang, Yuanlan Lu, Anyong Yu

**Affiliations:** Department of Emergency, Affiliated Hospital of Zunyi Medical University, Zunyi, Guizhou, China; Department of Cardiology, The First People’s Hospital of Zunyi, Zunyi, Guizhou, China

**Keywords:** organophosphorus pesticide poisoning, intestinal necrosis, exploratory laparotomy

## Abstract

**Objectives:**

Acute organophosphorus pesticide poisoning (AOPP) constitutes a common yet life-threatening emergency, particularly prevalent in rural regions of Asia. The condition results from inhibited cholinesterase activity, which induces acetylcholine accumulation at neural synapses. Clinically, its manifestations are categorized into muscarinic, nicotinic, and central types. Among these, mortality typically arises from respiratory paralysis, while neurological sequelae are frequently encountered. Nevertheless, reports of AOPP-induced intestinal necrosis remain exceedingly uncommon both domestically and internationally.

**Case presentation:**

In the present case, the patient experienced acute poisoning following oral ingestion of a high dose of dichlorvos. Despite initial standardized management, including atropine administration, pralidoxime therapy, and hemoperfusion, complications such as toxic encephalopathy and multiple organ dysfunction ensued. During hospitalization, acute peritonitis developed, and abdominal computed tomography revealed intestinal perforation accompanied by peritonitis. Exploratory laparotomy confirmed small intestinal necrosis and perforation of the ascending colon, necessitating resection of the necrotic segment and enterostomy. Postoperatively, the patient was transferred to the emergency intensive care unit for comprehensive supportive treatment and subsequently achieved full recovery before discharge.

**Conclusions:**

This case highlights that in patients with severe organophosphorus poisoning, any unexplained alteration in abdominal signs should prompt high clinical suspicion of gastrointestinal complications, and prompt surgical intervention may be lifesaving. However, as a single case report, this study carries inherent limitations, and future large-scale investigations remain essential to further substantiate these findings and develop more effective therapeutic strategies to enhance the overall management of AOPP.

## Background

Acute organophosphorus pesticide poisoning (AOPP) constitutes a major clinical and public health concern across rural regions of Asia, accounting for approximately two-thirds of suicide-related fatalities [1]. The primary toxic mechanism involves inhibition of cholinesterase activity, resulting in excessive accumulation of acetylcholine at nerve terminals and subsequent manifestation of muscarinic symptoms (e.g., increased airway secretions, abdominal pain, diarrhea, miosis, lacrimation, and salivation), nicotinic symptoms (e.g., facial, limb, and generalized skeletal muscle fasciculations), and central nervous system disturbances (e.g., dizziness, headache, delirium, and coma) [2]. Globally, particularly within developing nations, AOPP contributes to tens of thousands of deaths annually [2], 3]. The elderly population, characterized by age-related physiological decline and multiple comorbidities, represents a high-risk cohort for AOPP. Following exposure, clinical presentations often become increasingly complex, accompanied by heightened vulnerability to progression toward multiple organ dysfunction syndrome (MODS) [4]. In addition to frequently encountered complications such as respiratory failure and toxic encephalopathy, gastrointestinal manifestations, including stress ulcers, perforation, or necrosis, though infrequent, are associated with exceptionally high mortality. These gastrointestinal events are frequently obscured by the toxicological process itself, thereby complicating timely diagnosis [4], [5], [6], [7]. Therefore, an in-depth understanding of AOPP epidemiology and clinical features, particularly in elderly patients, bears substantial clinical significance. Early identification and intervention remain indispensable for optimizing prognosis. This report describes an elderly AOPP patient who developed concurrent toxic encephalopathy, gastrointestinal perforation, and MODS, and subsequently underwent successful surgical intervention, emphasizing the pivotal role of surgical management when conservative therapy proves ineffective. The case offers valuable clinical reference for managing severe AOPP-related complications and provides insight into diagnostic and therapeutic approaches applicable to comparable complex conditions [8], 9]. Through meticulous analysis of this rare presentation, clinicians’ awareness and management capabilities concerning multiple complications of AOPP may be enhanced, thereby reinforcing vigilance and improving therapeutic proficiency in addressing such uncommon but life-threatening conditions, ultimately contributing to improved outcomes among critically ill patients.

This study aimed to investigate the clinical characteristics and management strategies of acute organophosphorus pesticide poisoning complicated with intestinal necrosis. We hypothesized that early identification of abdominal complications and timely damage control surgery could improve the prognosis of severe AOPP patients. The research question was: how to effectively diagnose and treat intestinal necrosis/perforation as a rare but fatal complication in severe AOPP patients?

## Case presentation

A 73-year-old male patient was admitted to a local county hospital approximately 2 h after ingesting approximately 400 mL of dichlorvos. The patient had no notable previous medical history. Upon admission, physical examination revealed a toxic comatose condition, with a temperature of 36.3 °C, pulse rate of 109 beats/min, respiratory rate of 24 breaths/min, and blood pressure of 98/76 mmHg. Both pupils measured approximately 1 mm in diameter and exhibited a sluggish light reflex. Auscultation of the lungs revealed coarse breath sounds bilaterally, accompanied by scattered moist rales. Cardiac and abdominal examinations were unremarkable. The preliminary diagnosis was AOPP.

After admission, comprehensive treatment was promptly initiated, including repeated gastric lavage, catharsis, intravenous atropine administration (cumulative dose 20 mg), intravenous infusion of pralidoxime (2.0 g), hemoperfusion, and mechanical ventilation support. Despite these interventions, the patient’s consciousness did not improve after five consecutive days of standardized treatment at the local hospital, and acute peritonitis manifestations emerged on day 5 after poisoning, characterized by pronounced abdominal distension, muscular rigidity, and absence of bowel sounds. The patient was subsequently transferred to this hospital for further management on day 5 after poisoning. On admission, physical examination revealed a temperature of 37.8 °C, pulse rate of 129 beats/min, respiratory rate of 19 breaths/min, and blood pressure of 89/65 mmHg. Bilateral pupils were equal in size, approximately 3 mm in diameter, and exhibited brisk light reflexes. Auscultation of both lungs revealed coarse breath sounds accompanied by rales. Cardiac rhythm was regular, with no murmurs detected over any valvular area. The abdomen appeared distended with generalized muscular rigidity, tenderness, and rebound tenderness, while bowel sounds were absent. Mottled skin (grade 3) was noted on both lower extremities, and bladder pressure was measured at 22 cmH_2_O. Laboratory investigations indicated a cholinesterase level of 1.0 kU/L (reference range: 4.6–11.5 kU/L) and a creatinine concentration of 346 μmol/L. Toxicological analysis identified dichlorvos (35.6 ng/mL) and trimethyl phosphate (130.8 ng/mL) in the blood. Both compounds were also detected in gastric fluid and urine samples. Chest computed tomography (CT) revealed bilateral pneumonia with small pleural effusions. Abdominal CT was performed immediately after admission to our hospital (day 5 after poisoning) and demonstrated peritonitis accompanied by multiple dilated bowel loops containing gas and fluid. The small intestine in the right lower quadrant exhibited irregular morphology with localized gas-density shadows, suggestive of regional inflammation or perforation (Figure 1). Emergency exploratory laparotomy was performed on the same day of admission (day 5 after poisoning). Intraoperatively, segmental necrosis of the small intestine was observed approximately 100 cm proximal to the ileocecal junction, together with two perforations in the ascending colon measuring approximately 1.0 and 0.8 cm in diameter, respectively. Resection of the necrotic bowel segment (approximately 60 cm) and ileostomy were subsequently performed (Figure 2). Histopathological examination of the resected intestinal tissue demonstrated hemorrhagic infarction with full-thickness acute suppurative inflammation (Figure 3). Postoperatively, the patient was transferred to the emergency intensive care unit (EICU) for continued monitoring and management, including anti-infective therapy, nutritional support, and maintenance of airway function. After a course of intensive treatment, vital signs stabilized by postoperative day 10, intestinal function was partially restored, and the patient was discharged after one month of rehabilitation.

**Figure 1:**
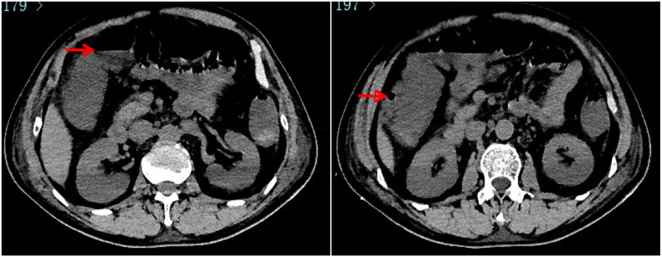
Abdominal CT imaging following poisoning, primarily showing extensive intestinal distension, gas accumulation, and fluid retention, with scattered intraluminal air-density shadows suggesting localized inflammation or perforation.

**Figure 2:**
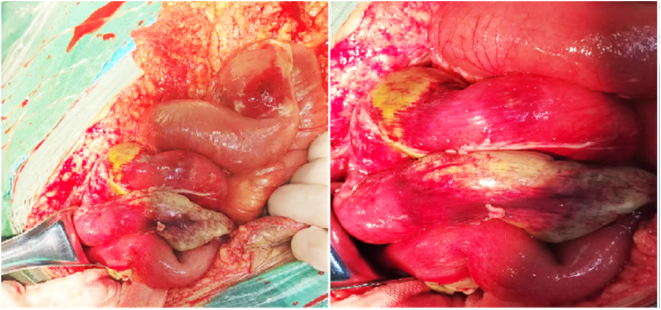
Gross specimen of the resected intestinal segment obtained during surgery, revealing areas of dark red discoloration and loss of surface luster consistent with necrosis; the non-necrotic segments also display marked edema and thickened intestinal walls.

**Figure 3:**
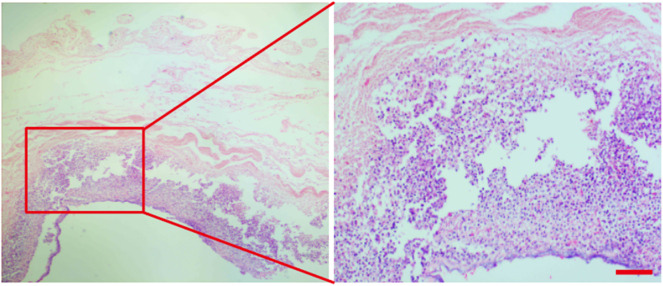
Histopathological findings demonstrating characteristic intraoperative intestinal alterations, including hemorrhagic infarction of the small intestine and transmural acute suppurative inflammation, consistent with changes resulting from severe ischemia–infection interaction.

## Discussion

In the present study, we found that severe acute organophosphorus pesticide poisoning can lead to rare but life-threatening intestinal necrosis and perforation, and early diagnosis combined with damage control surgery can significantly improve patient survival. AOPP is a frequent critical emergency encountered in emergency departments, where clinicians are well acquainted with its characteristic muscarinic, nicotinic, and central nervous system manifestations. Nevertheless, the complications resulting from AOPP extend far beyond the acute cholinergic crisis, involving complex pathophysiological processes that may induce systemic inflammatory responses, oxidative stress, and progression to MODS [2]. In the present case, a 73-year-old male who ingested a large dose of dichlorvos underwent standardized medical detoxification, blood purification, and organ function support. However, his condition continued to deteriorate. Ultimately, the predominant clinical manifestation was intestinal necrosis and perforation, representing a rare form of surgical acute abdomen. The successful management of this case offers valuable reference for advancing the understanding of occult complications in severe AOPP and for exploring the multidisciplinary treatment (MDT) approach [10]. This report provides an in-depth discussion focusing on the distinct features of this case, underlying pathogenic mechanisms, diagnostic and therapeutic decision-making, and its broader clinical significance.

Among the numerous complications associated with AOPP, gastrointestinal perforation or necrosis is rarely documented and can easily be obscured by severe gastrointestinal manifestations such as vomiting, diarrhea, and toxic ileus caused by the poisoning itself [3], 11]. Most available literature has concentrated on the impact of AOPP on organs, including the heart, lungs, and brain, whereas limited attention has been directed toward the severe intestinal injury, which functions as both an “initiating organ” and a “target organ”. In this case, five days after poisoning and during comprehensive therapy, the patient developed progressively worsening abdominal distension, muscular rigidity, and absence of bowel sounds. Abdominal CT revealed marked intestinal dilatation with fluid retention and signs indicative of localized perforation. Intraoperative findings confirmed segmental small bowel necrosis and perforation of the ascending colon. This body of evidence clearly indicates that intestinal ischemic necrosis, in contrast to toxic ileus, constitutes a progressive organic lesion. A review of the literature reveals that reports of AOPP-related gastrointestinal perforation are primarily confined to the stomach and duodenum [3], 5]. Cases involving concurrent lesions of both the small intestine and colon, as seen in this patient, are exceedingly uncommon. These findings imply that gastrointestinal injury caused by AOPP may be extensive and lack organ specificity.

Intestinal necrosis has multiple etiologies, including mechanical obstruction, arterial embolism, venous thrombosis, and non-obstructive disorders induced by various factors [12]. Its pathogenesis is generally associated with intestinal vascular injury, which may result from edema caused by intestinal wall distension and obstruction or from hemodynamic disturbances directly triggered by vascular occlusion. The mechanism underlying organophosphate-induced intestinal necrosis remains incompletely understood. Excessive accumulation of acetylcholine following organophosphate exposure overstimulates muscarinic receptors, inducing intestinal hypermotility through cyclic guanosine monophosphate/protein kinase G-dependent signaling pathways [13], 14], while simultaneously promoting the release of adrenaline and noradrenaline via nicotinic receptor activation, thereby leading to intestinal vasospasm and microcirculatory disruption [15]. The occurrence of intestinal necrosis in this patient was not attributable to a single mechanism but rather resulted from the cumulative and synergistic effects of multiple pathophysiological processes. Dichlorvos, ingested in large quantities, is a highly toxic organophosphate pesticide whose parent compound and metabolites possess potent mucosal corrosive properties. Ingestion of a large dose can directly injure the mucosal barrier of the digestive tract from the esophagus to the colon, producing chemical burns, erosions, and even ulcers, thus establishing an anatomic basis for subsequent perforation [4], 16]. Moreover, the accumulation of acetylcholine in AOPP induces systemic vasomotor dysfunction. During the early stage of poisoning, muscarinic stimulation may lead to visceral vasodilation and congestion. However, as the intoxication progresses, persistent inflammatory responses and microcirculatory impairment ultimately cause constriction of the intestinal vascular bed and a marked decline in perfusion [6]. The hypotensive state observed during the course of this illness further aggravated intestinal ischemia. Consequently, this combined insult of “corrosion and ischemia” initiates the first stage of intestinal necrosis. AOPP itself can elicit a robust inflammatory cascade. After poisoning, massive release of pro-inflammatory cytokines (e.g., TNF-α, IL-6, IL-1β) into the circulation provokes systemic inflammatory response syndrome [7]. The intestine, as the body’s largest bacterial reservoir and toxin source, undergoes severe compromise of mucosal barrier integrity under ischemic and inflammatory stress, facilitating bacterial and endotoxin translocation into the portal and systemic circulation. This process leads to “gut-derived sepsis”, which accelerates the progression of MODS (Figure 4) [8]. Furthermore, during clinical resuscitation and hemoperfusion, intestinal blood flow may be partially restored. However, subsequent oxidative stress and reperfusion injury generate large quantities of oxygen-free radicals, resulting in a “secondary insult” to already damaged intestinal cells and promoting apoptosis and tissue necrosis [9]. The development of secondary pneumonia and renal dysfunction (creatinine 346 μmol/L) in this patient represents clinical manifestations of MODS, with the intestine likely serving as the central driving organ in this vicious cycle. During AOPP resuscitation, administration of large doses of atropine, though essential for survival, may also produce adverse effects. While atropine inhibits glandular secretion and relieves smooth muscle spasm, it simultaneously suppresses gastrointestinal motility, predisposing to stagnation of intestinal contents and elevated intraluminal pressure. On the background of preexisting intestinal wall injury, these effects further heighten the risk of perforation [17]. Additionally, advanced age compounded the pathophysiological insult. This elderly patient exhibited diminished vascular elasticity, reduced tissue repair capacity, and attenuated immune responsiveness compared with younger individuals. Such age-related physiological decline limited compensatory mechanisms against the aforementioned dual insults, ultimately predisposing to extensive tissue necrosis. Therefore, advanced age represents an independent risk factor for severe complications in AOPP.

**Figure 4:**
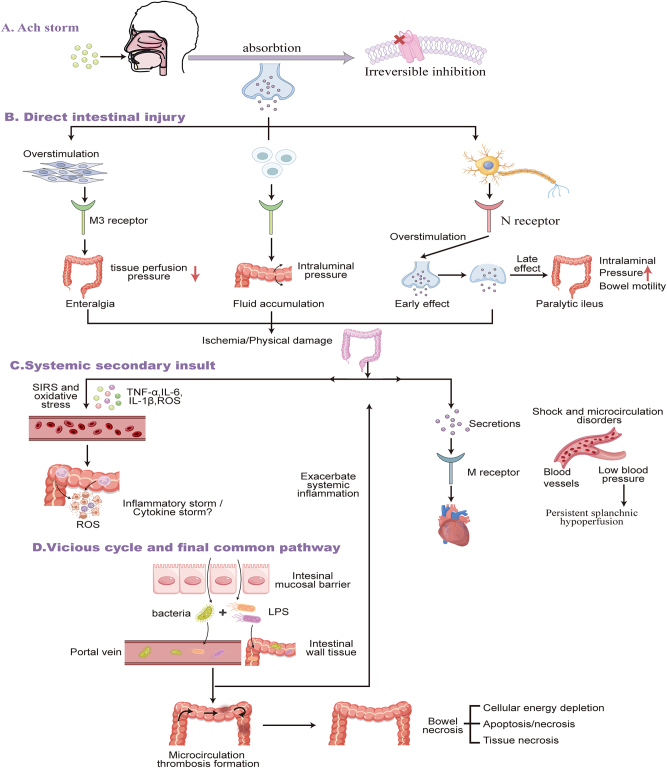
Schematic illustration of the proposed pathophysiological mechanism of intestinal necrosis induced by AOPP. This diagram depicts the primary mechanisms through which AOPP induces intestinal necrosis, encompassing toxin-mediated excessive inflammatory responses and oxidative stress, subsequent impairment of the intestinal barrier, translocation of bacterial flora and endotoxins, and ultimately aggravation of systemic injury.

Another key determinant of the successful outcome in this case was the clinicians’ ability to transcend the conventional notion that “all symptoms in AOPP arise solely from poisoning”, maintaining heightened vigilance toward newly emerging abdominal signs that could not be explained by the toxic process itself. In patients with severe AOPP, particularly those with impaired consciousness, classical manifestations of peritonitis such as abdominal pain and muscular rigidity are frequently obscured or misinterpreted as toxic ileus, leading to missed opportunities for timely surgical intervention [18], [19], [20]. When deterioration of the patient’s abdominal findings was observed, the medical team in this case did not rely exclusively on physical examination but promptly utilized abdominal CT as an essential diagnostic modality. The CT images demonstrated marked intestinal distension and ascites, with indistinct intestinal wall margins in the right lower quadrant and suspected free air, thereby providing compelling objective evidence for performing emergency exploratory laparotomy. These findings clearly highlight the pivotal diagnostic value of modern imaging techniques in differentiating causes of acute abdomen [21].

Previous reports of AOPP complicated with intestinal injury have mainly focused on gastric or duodenal perforation, with only a few cases describing small intestinal necrosis [3], 5], 21]. To our knowledge, this is one of the few reports of severe AOPP simultaneously presenting with small intestinal necrosis and ascending colon perforation. Similar to previous literature, our case confirmed that severe AOPP can cause severe gastrointestinal ischemic necrosis due to direct toxin corrosion, microcirculatory disturbance, and systemic inflammatory response [21]. However, different from most previous reports, our patient achieved complete recovery through timely damage control surgery (resection + enterostomy) rather than conservative treatment or primary anastomosis. This suggests that enterostomy may be more suitable for critically ill patients with severe systemic inflammatory response and multiple organ dysfunction, which is consistent with the principle of damage control surgery in acute abdomen [22], [23], [24].

Currently, no established guidelines or consensus exist specifically for the management of AOPP complicated by intestinal perforation. Nevertheless, according to general principles governing the treatment of sepsis and gastrointestinal perforation, the concept of “damage control surgery” holds paramount significance [22], [23], [24]. In this case, primary intestinal anastomosis was deliberately avoided. Instead, a decisive strategy of “resection of necrotic bowel and enterostomy” was implemented, constituting a highly judicious surgical decision. This approach not only enabled rapid control of intra-abdominal contamination and halted the ongoing progression of sepsis but also circumvented high-risk anastomosis during the peak of the patient’s systemic inflammatory response, thereby minimizing the likelihood of anastomotic leakage. This operative strategy afforded valuable time for subsequent organ support in the EICU and served as the foundation for the patient’s eventual recovery. Admittedly, as a single case report, this study possesses inherent limitations. The generalizability of its conclusions remains constrained, and the incidence, risk factors, and optimal timing of this complication require further verification through large-scale clinical investigations.

## Conclusions

Severe AOPP, particularly in elderly patients, may be complicated by rare occurrences of intestinal necrosis and perforation. The underlying pathogenesis involves the synergistic interaction of multiple factors, including direct pesticide toxicity, intestinal ischemia, and systemic inflammatory response. Clinicians should transcend conventional perspectives and maintain heightened vigilance toward evolving abdominal signs. When surgical complications are suspected, imaging modalities should be promptly employed to achieve early and definitive diagnosis, followed by timely surgical intervention guided by the principle of “damage control”. Close MDT collaboration constitutes the foundation of successful management in these critically ill patients. This case offers a valuable reference for improving clinical recognition and management of this uncommon complication. Further investigations are warranted to elucidate its epidemiological features, potential predictive biomarkers, and standardized diagnostic and therapeutic strategies.
